# Combining Diagnostic Imaging and Pathology for Improving Diagnosis and Prognosis of Cancer

**DOI:** 10.1155/2019/9429761

**Published:** 2019-07-01

**Authors:** Orazio Schillaci, Manuel Scimeca, Nicola Toschi, Rita Bonfiglio, Nicoletta Urbano, Elena Bonanno

**Affiliations:** ^1^Department of Biomedicine and Prevention, University of Rome “Tor Vergata”, Via Montpellier 1, Rome 00133, Italy; ^2^IRCCS Neuromed, Pozzilli, Italy; ^3^University of San Raffaele, Via di Val Cannuta 247, 00166 Rome, Italy; ^4^Fondazione Umberto Veronesi (FUV), Piazza Velasca 5, 20122 Milano, Italy; ^5^Martinos Center for Biomedical Imaging, Boston, MA, USA; ^6^Harvard Medical School, Boston, MA, USA; ^7^Department of Experimental Medicine, University “Tor Vergata”, Via Montpellier 1, Rome 00133, Italy; ^8^Nuclear Medicine, Policlinico “Tor Vergata”, Rome, Italy; ^9^IRCCS Neuromed Lab, “Diagnostica Medica”, “Villa dei Platani”, Avellino, Italy

## Abstract

In the era of personalized medicine, the management of oncological patients requires a translational and multidisciplinary approach. During early phases of cancer development, biochemical alterations of cell metabolism occur much before the formation of detectable tumour masses. Current molecular imaging techniques, targeted to the study of molecular kinetics, employ molecular tracers capable of detecting cancer lesions with both high sensitivity and specificity while also providing essential information for both prognosis and therapy. On the contrary, complementary and crucial information is provided by histopathological examination and ancillary techniques such as immunohistochemistry. Thus, the successful collaboration between diagnostic imaging and anatomic pathology can represent a fundamental step in the “tortuous” but decisive path towards personalized medicine.

## 1. Introduction

Molecular imaging is quickly gaining importance in both biomedical research and clinical diagnostics. During the early phases of cancer development, biochemical alterations of cell metabolism occur before the formation of detectable tumour masses. Current molecular imaging techniques, targeted to the study of molecular kinetics, employ molecular tracers capable of detecting cancer lesions with both high sensitivity and specificity while also providing essential information for both prognosis and therapy. On the contrary, crucial information is provided by histopathological examination and ancillary techniques such as immunohistochemistry. Indeed, in situ analysis of cancer biomarkers by immunohistochemistry provides prognostic and predictive information related to the metabolic characteristics of the tumour mass. Therefore, a multidisciplinary approach based on the collaboration between diagnostic imaging and anatomic pathology could improve the management of oncological patients, further supporting the current goal of a completely personalized medicine approach in oncology. In detail, this new approach will allow clinical doctors to investigate the living body to identify disease, monitor progression, or treat medical conditions at a molecular level [1]. In this review, we discuss the possible alliance between diagnostic imaging and pathology with a focus on teamwork between nuclear medicine and anatomic pathology.

## 2. Diagnostic Imaging and Anatomic Pathology: An Alliance for the Diagnosis of Oncological Patients

The use of diagnostic imaging has increased steadily over the past decade, dramatically contributing to improved management of oncological patients. Indeed, high-resolution images provided by computed tomography (CT), magnetic resonance imaging (MRI), and ultrasound (US) allow early detection of a vast number of tumour masses. In this context, combined imaging based on positron-emission tomography- (PET-) CT plays a central role in the diagnosis, staging, and posttreatment follow-up of patients with neoplastic diseases [2], and several studies have shown the increase in diagnostic value resulting from combined PET-CT. For instance, Lardinois et al. reported for the first time the advantage of PET-CT over other techniques for TNM staging [3]. In addition, recent studies have shown that PET-CT is the best imaging modality for tumour staging and specifically that it is superior both to PET and to PET and CT performed separately [4]. Combined PET-CT acquisition can therefore improve the diagnostic value of the examination and implements the concept of “one-stop shop” recently introduced in modern clinical medicine, which can also be extended to more recent PET-MR developments (see below) [5]. The possibility of performing TNM staging and formulating a full diagnosis with a single examination also drastically reduces the time commonly required to perform all diagnostic examinations separately [2].

**Figure 1:**
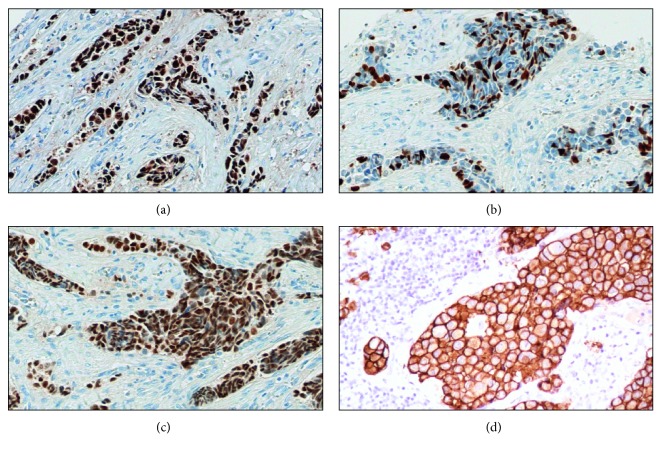
Evaluation of in situ breast cancer biomarkers. Image showing (a) Ki67 nuclear expression in an infiltrating breast cancer, (b) expression of ER in an infiltrating breast cancer, (c) PR expression in an infiltrating breast cancer, and (d) HER2 expression (score 3) in an infiltrating breast cancer.

While radiography, ultrasonography, and CT are the main imaging techniques employed in cancer diagnosis, MRI is an emerging imaging diagnosis method that, while well established in the clinical practice, is in continuous development. Specifically, MRI can produce three-dimensional, multimodal images in a noninvasive way, without the use of ionizing radiation and with exceptional spatial and contrast resolution, allowing unprecedented accuracy in tumour investigation [6]. The novelty of MRI analysis is focused mainly on improving the anatomical resolution and on the advent of functional as well as molecular approaches [7]. Currently, available MRI techniques allow both structural assessment and evaluation of different physiopathological processes of the tumour microenvironment [7, 8]. Also, structural and functional MRI results in a more comprehensive evaluation of the extension and activity of neoplastic diseases [9]. Of note, a correct oncologic status evaluation allows for the establishment of better therapeutic strategies, with a favourable impact on prognosis as well as survival. In addition, the use of perfusion, spectroscopy, and diffusion MRI modalities can provide essential data about angiogenesis, cell metabolism, and cellularity of cancer lesions [10–12]. Still, histopathological studies are essential to validate the results of PET-CT and MRI exams [13]. Analysis of biopsies or surgical specimens still represents the gold standard for the diagnosis and full characterization of neoplastic lesions [14]. Moreover, the introduction of novel and high-performance molecular biology techniques on paraffin sections allows to compare metabolic data of molecular imaging (i.e., uptake of FDG or radiolabelled antibodies) with the in situ localization of molecules involved in cancer pathways. As concerns the relationship between MRI and histopathological data, breast cancer in particular offers important perspectives. Indeed, several studies have focused on the identification of MRI features that can predict the molecular patterns of breast cancer such as ER, PR, Ki67, and HER2 status [15]. In this context, Ouk et al. reported that triple-negative breast cancer (TNBC) was larger and better defined and had more necrotic tissue compared to other cancers and that necrosis yielded high T2-weighted signal intensity and apparent diffusion coefficient (ADC) values [16]. Uematsu et al. found similar MR features for TN cancers, including high intratumoural signal intensity on T2-weighted MR imaging and intratumoural necrosis [17]. Bae et al. reported that, in MRI, androgen-receptor-positive TN cancers were more likely to be associated with nonmass types and a higher incidence of irregular and spiculated lesions than androgen-receptor-negative TN cancers [18]. Also, a recent study demonstrated a correlation between HER2 overexpressed cancers and persistent enhancement in the delayed phase on MRI, suggesting that MRI can be potentially useful in the diagnosis and subtyping of breast cancer. The information obtained by immunohistochemical analysis can generate greater concordance when compared with molecular imaging techniques such as PET and SPECT (single-photon-emission computerized tomography). In this scenario, nuclear medicine and anatomic pathology share several common interests. They constitute two fundamental approaches for the establishment of diagnosis, clinical monitoring, prognosis, and therapy response. Both disciplines are similarly interested in finding anatomic lesions and in deepening knowledge about their nature at a cellular and molecular level [19, 20]. More than ever before, nuclear imaging is playing an integral role during both initial investigations and follow-up of patients with acute and chronic illnesses. It comprises several techniques, such as PET and SPECT, that use specific radiopharmaceuticals to image certain molecular interactions belonging to biological processes in vivo and are therefore able to deliver information about biomarker expression, tumour burden, tumour metabolism, receptor status, and proliferation. In particular, the spatial resolution of PET-CT offers exceptional insights into the human body that enable physicians to identify and characterize tumoural lesions in the early stages of their development [21–23]. It is important to note that, in some cases, it is possible to use the same antibodies to detect target molecules both *in vivo* by immuno-PET analysis and *ex vivo* by immunohistochemistry [24]. The treatment of breast and prostate cancers is based on immunohistochemical analysis of well-known prognostic markers such as ER, PR, Ki67, cerb2 (Figure 1), or PSA [25, 26]. Nevertheless, cancer cells frequently undergo selection during pharmacological treatment, hence possibly changing their dominant molecular phenotype and leading to drug resistance. Even though heterogeneous temporal evolution of cancer cells can be evaluated through sequential biopsies, this would imply the necessity of repeated invasive procedures. To reduce the number of biopsies and simultaneously increase the patient's quality of life, PET radiolabelled molecules directed against markers which are prognostic and predictive in cancer (i.e., radiolabelled anti-cerb2 antibodies [27] or radiolabelled PSMA inhibitors [28]) have been developed. In the last years, several studies identify some significant correlations among histological parameters, immunohistochemical data, and ^18^F‐FDG uptake (PET‐MRI analysis) in patients affected by head or neck squamous-cell carcinoma [29, 30]. All at once, these data can lay the foundation for the development of new *in vivo* analysis capable of predicting prognosis and therapy response early without the support of histological analysis. Specifically, histological samples offer the possibility to characterize cancer lesions from a genetic point of view, by studying chromosomal alterations like gene amplification or DNA fragment translocation [31], and at an ultrastructural and atomic level, thanks to the performance of scanning and transmission electron microscopes and their ancillary technologies (such as energy-dispersive X-ray microanalysis and immunogold labelling) [32–35]. These new approaches may be considered a further element capable of “linking” molecular imaging and anatomic pathology. A great proof of nuclear medicine and pathology collaboration is the characterization of breast microcalcification. Indeed, recent studies, focused on exploring the clinical and biological significance of breast microcalcifications, demonstrated that their elemental composition and histological aspect were related to patients' prognosis [36, 37]. In addition, in these studies, it was shown that microcalcifications made of hydroxyapatite are actively produced by cancer osteoblast-like cells (OLCs) in a process similar to what occurs during physiological bone matrix formation [38–40]. On the basis of this evidence, molecular imaging could be used to discriminate cancer from any other type of lesion by studying the metabolic activity of breast tissues associated with the presence of microcalcifications as shown by Ahrens et al. in a mouse model [41].

The affinity between nuclear medicine- and anatomic pathology-based diagnostics can therefore lay the basis for the creation of a synergic framework at the service of personalized medicine.

## 3. Digital Pathology

Since the 1980s, radiology has been revolutionized by the introduction of digital imaging, with a resulting improvement in quality and safety of reporting as well as disrupting innovation in the analysis and manipulation of radiological images [42]. Also, the recent introduction of digital pathology and whole slide imaging (WSI) (i.e., the complete digitization of slides) in anatomic pathology is transforming the practice of histodiagnostics [43]. During the last few decades, the introduction of digital cameras producing still images and microscope-mounted video cameras that allow live examination of slides has radically changed optical pathology [44]. In addition, approximately a decade ago, further improvements of these techniques resulted from the creation of digital slide scanners. These still or dynamic images can be transferred to remote sites to be assessed by another pathologist—that is, what is commonly called telepathology [45, 46]. This has facilitated applications such as teleconsultation and frozen section diagnosis [47].

WSI produces high-resolution digital images and involves relatively high-speed digitization of glass slides of different samples (e.g., tissue sections, smears, and thin-layer cytology), scanning them at multiple magnifications and focal planes (*x*, *y*, and *z* axes) [48]. Compared to static and live digital images, WSI is generally more beneficial. For educational and training purposes, WSI is more interactive and easy to share and provides access to the entire slide to help answer “on-the-spot” clinical questions at tumour boards. One can also generate teaching sets (virtual slide boxes) that can include a wide case range as well as rare cases that do not fade, break, or disappear. The digital imaging process includes four key steps: (a) image acquisition or capture; (b) storage and management, manipulation, annotation, and editing; and (c) viewing, displaying, or sharing of images. From a clinical point of view, digital images can be used for primary diagnosis, second opinions, telepathology, quality assurance (e.g., re-review and proficiency testing), archiving and sharing, training, education and conferencing, image analysis, research and publications, and marketing and business purposes, as well as tracking. Unfortunately, to date, the operational phases of digital pathology have not yet been standardized. Before digital slides are widely used for routine clinical analysis, standard procedures are needed and the entire imaging process is validated through large-cohort studies. However, in 2016, García-Rojo [49] released the first document with the guidelines and position papers from the Canadian Association of Pathologists, the College of American Pathologists, the American Telemedicine Association, the Digital Pathology Association, the Food and Drug Administration, the Centers for Medicare and Medicaid Services, the Centers for Disease Control and Prevention, the Society of Toxicologic Pathology, the European Commission, the Spanish Society of Anatomic Pathology, the Royal College of Pathologists, and the Royal College of Pathologists of Australasia.

Still, automated image analysis will enhance diagnostic efficacy in histopathology. Because inspection of WSI is probably slightly more time-consuming than that of conventional slides [50], the creation of software packages for detecting regions of interest will be advantageous and speed up the workflow, especially if those areas of interest could be outlined before the pathologist sees the image. To this end, grid computing would probably be needed to be able to apply several algorithms to WSI [51]. Still, software for computerized quantification of immunohistochemically stained WSI to improve the objective assessment of the immunoreactivity is already available from several scanner vendors. Such software estimates colour intensity relative to control cells and has Food and Drug Administration regulatory approval in the United States for the quantification of nuclear markers such as estrogen receptor (ER) or cell membrane markers such as HER2. Notably, the treatment of breast cancer patients is often based on immunohistochemical analysis of ER, progesterone receptor (PgR), Ki67, and HER2 [42, 52] (Figure 1).

**Figure 2:**
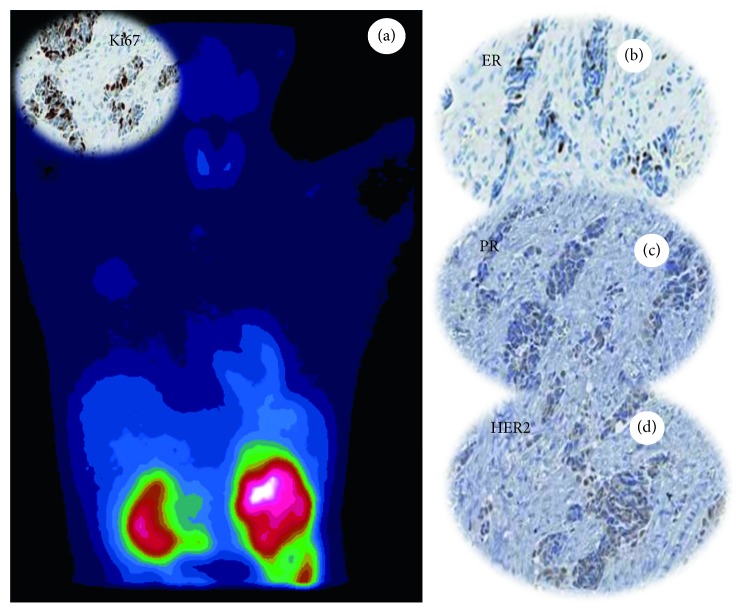
99mTc sestamibi SPECT analysis and histological evaluation of a triple-negative breast cancer. (a) Image showing the uptake of sestamibi in a cancer lesion characterized by a high proliferation rate (Ki67). Immunohistochemical analysis displaying triple-negative phenotypes of breast lesions: (b) ER negative; (c) PR negative; (d) HER2 score 0.

**Figure 3:**
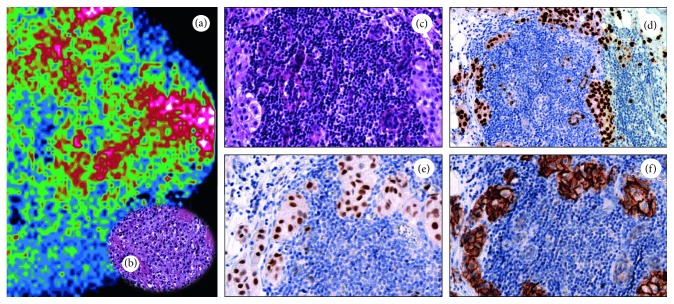
99mTc sestamibi SPECT analysis and histological evaluation in a breast cancer patient. (a) Image showing sestamibi uptake in a breast cancer patients. (b) Histological evaluation (H&E) of the breast biopsy of the patient subjected to 99mTc sestamibi SPECT. (c) Sentinel lymph node with macrometastasis. Immunohistochemical analysis of lymph nodes displaying triple-positive phenotypes of breast cancer cells: (d) ER positive; (e) PR positive; (f) HER2 score 3.

Despite that new technologies open the possibility to detect digital spatial profiling of proteins and RNA in fixed tissue [53], currently diagnostic immunohistochemical analysis cannot capture the known spatial heterogeneity of breast cancer defined at the intratumoural, intrametastatic, and intermetastatic levels. Thus, the quantification of immunohistochemical data can be used to design new molecular diagnostic imaging or therapeutic protocols (PET- or SPECT-CT) that improve the patient's quality of life by (a) reducing the number of biopsies, (b) increasing the accuracy of the diagnosis, and (c) identifying in “real time” the molecular transformation of cancerous cells. In particular, the use of radiolabelled molecules capable of quantifying the expression of ER (i.e., 16*α*-^18^F-fluoro-17*β*-estradiol) or cerb2 (i.e., radiolabelled anti-HER2 antibodies) by PET-CT could represent an alternative and reliable method for the management of breast cancer patients [54]. Our recent studies highlighted the possible role of 99mTc sestamibi SPECT as a possible tool for early detection of breast cancer lesions with high bone metastatic potential (Figures 2 and 3) [40, 55]. In particular, we showed a putative correlation between 99mTc sestamibi uptake and aggressiveness of the tumours. High sestamibi uptake was detected in both triple-negative breast cancer lesions (Figure 2) and poorly differentiated infiltrating breast carcinomas forming lymph node (Figure 3) and bone metastasis.

**Figure 4:**
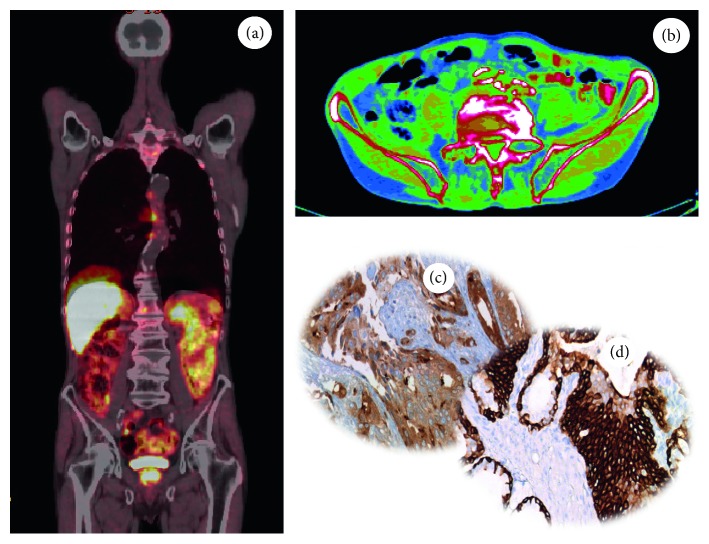
^18^F-choline PET analysis and histological evaluation in a prostate cancer patient. Images showing (a, b) the uptake of ^18^F-choline in a prostate cancer lesion, (c) PSA expression in a prostate cancer biopsy, and (d) ck34be12 expression in a prostate cancer.

Similarly, the search for new biomarkers of prostate cancer is constantly advancing. The most important radiotracer used for diagnosis and staging of prostate cancer is choline (^11^C-choline and ^18^F-choline) (Figure 4) [56]. Currently, choline PET and PET-CT techniques deliver high sensitivity and specificity for the diagnosis of locoregional and distant metastases in prostate cancer patients with recurrence of disease [56]. In our experience, a high ^18^F-choline signal is significantly associated with PSA plasma levels, histological grading, and in situ PSA expression (Figure 4). The comparative expression of PSMA on prostate biopsies analyzed through digital pathology and molecular imaging techniques can further improve the accuracy of nuclear medicine analysis by providing elements for both identification of new PSMA inhibitors and comprehension of biological mechanisms related to PSMA expression in prostate lesions. In this context, Bernacki et al. demonstrated that anti-PSMA immunohistochemical analysis is a promising marker for the identification of metastatic prostate carcinomas in surgical specimens [57].

## 4. From Molecular Pathology to Molecular Imaging and *Vice Versa*

Molecular imaging is a rapidly developing translational discipline which involves molecular biology, chemistry, computer science, engineering, and medicine [58]. It can provide noninvasive and real-time visualization as well as quantification of physiological or pathological processes in the living organism at the cellular or molecular level.

Recently, diagnostic sciences have been rapidly changing under the influence of the concept of personalized medicine; thus, molecular diagnostic techniques are increasingly used for the identification of individualized therapy and prediction of treatment response [59]. As a direct consequence, the histopathology discipline is transforming into a molecular pathology science discipline. Due to recent technological developments, including mass spectrometry and different -omic approaches, conventional pathology techniques such as immunohistochemistry are at present frequently accompanied by genetic, epigenetic, and proteomic analyses [60]. These new technological approaches allow the identification of prognostically and therapeutically relevant in situ molecular targets that may represent a “priceless treasure” for the development of new diagnostic protocols based on molecular imaging [61]. Such modalities in general and PET-CT in particular may overcome the main limitations of cancer biopsies, i.e., the spatial and temporal tumour heterogeneities. In this context, it is known that the analysis of biopsy samples often underestimates the gene/protein alteration burden of primary tumours and that based on clonal evolution of cancer cells, the biology of metastases can significantly differ from that of the primary tumour [62–66]. In addition, several metastatic sites are difficult to access for biopsies [67]. In this context, several clinical and preclinical studies, based on histopathological evidence, have been carried out in order to set up molecular imaging protocols for early detection or therapy of human cancer. McKnight et al. developed a new PET radiotracer that simultaneously delineates both of EGFR and HER3 receptors in a mouse model of pancreatic cancer [68]. Histopathological and clinical investigations showed that these receptor tyrosine kinases are implicated in resistance to treatment and that their blockade stimulates compensatory pathways which can rescue signalling activity [69]. Thus, the dual immuno-PET analysis reported in this study could extend diagnostic possibilities for the cure of pancreatic carcinomas [70]. Also, in the field of immuno-PET, recent studies highlighted the possibility to quantify *in vivo* HER2 expression in breast cancer patients. It is known that HER2 overexpression is related with a worse survival in both node-positive and node-negative breast cancer patients [71]. In addition, HER2 expression represents a predictive indication for therapy with trastuzumab, a human monoclonal anti-HER2 antibody that has provided a distinct therapeutic advantage [72, 73] over other strategies. Already in 2005, Robinson et al. validated a method for using a clinical PET-computed tomography scanner to quantify tumour uptake of anti-HER2 antibodies and other HER2-targeting drugs [74]. This method has the potential to profoundly change the management of breast cancer patients—the selection of patients eligible for anti-HER2 therapy could be carried out by PET analysis. Also, molecular imaging analysis could be used to enhance the efficacy of immune-modulation therapies. In recent years, the use of anti-immune checkpoint antibodies has represented a new frontier in the care of several solid tumours such as lung, breast, and prostate tumours [75]. Specifically, therapies that target the PD-1/PD-L1 pathway have shown promising and inspiring effects with remarkably durable responses in lung tumours [76–79] and TNBCs [80]. Indeed, numerous Phase I/II clinical trials to test antibodies that target PD-1 or PD-L1 in breast cancer are ongoing [81, 82]. Several investigations displayed that low levels of PD-L1 in clinically relevant cancer can be imaged with immuno-PET using recombinant human anti-PD-L1 antibodies [83]. This approach can provide new hopes for cancer patients for which the common analyses did not allow to identify specific diagnostic protocols.

One of the more frequent joint imaging histopathological applications concerns the in situ expression of Ki67, the main immunohistochemical marker of cancer proliferation [84], and FDG-PET analysis. Unfortunately, an exact association between Ki67 expression and FDG uptake in human cancer has not yet been reliably identified. However, recent studies reported solid association between these data. In particular, FDG-PET analysis and Ki67 in situ evaluation showed strict correlation in the diagnosis of ovarian [85] and lung cancers [86, 87] and squamous-cell carcinomas of the head and neck [88–90].

In conclusion, the mutual support between diagnostic imaging and anatomic pathology can assist the oncologists in the choice of more appropriate therapeutic protocols both for primary lesions and for recurrence. Also, image-processing strategies can be used to develop artificial intelligence algorithms which have the potential to aid diagnosis, prognosis, and therapeutic decision-making by integrating large-scale, multimodal information and presenting it to the pathologist and oncologist in an interpretable manner.

## 5. Computer-Aided Diagnosis and Artificial Intelligence: New Perspectives for Diagnostic Imaging and Pathology Cooperation

While artificial intelligence (AI) in general and machine learning in particular have been increasingly used in studies presenting work applied to the biomedical sciences, during the last three or four years, one particular class of methods known as deep neural networks (DNNs) has provided disruptive innovation in a number of fields, including medical applications in general and decision support in complex problems based on multimodal data in particular. Of note, DNNs have had extremely high success rates in the field of oncology. Examples include using nuclear features to classify breast lesions [90], predicting survival in non-small-cell lung cancers [91], and identifying metastases [92]. DNN-based analyses will likely continue to push the boundaries of clinically relevant morphologic information not found in our current human-derived pathologic criteria [91]. The peculiarity of DNNs lies in the ability to naturally deal with images by a number of strategies including repeated filtering, decomposition, and reaggregation into high-order features which can represent diagnostic information initially unidentifiable to the human eye. With enough training, a DNN can discern extremely subtle clues and local alterations in medical images, and the trained model can be used to classify “unseen,” future patients [91]. The capabilities of DNNs can, for example, also be extended to predicting molecular alterations (e.g., genetic changes) as long as the training data have been annotated both clinically and genomically in an accurate manner [91]. Given the joint potential of nuclear imaging along with WSI to produce a wealth of data whose minute details cannot be integrated by a human observer in a systematic way, DNNs offer a unique opportunity to also generate important savings in terms of cost when faced with the decision of when molecular testing needs to be prioritized in so-called precision oncology. Pathology work could also be significantly enhanced by training DNNs to emphasize and highlight morphological signs whose identification is time-consuming to the naked eye but may result in diagnostically actionable items (e.g., lymphovascular invasion or mitotic figures) [92].

This type of workflow would not only render pathology and imaging work quickly and more accurately but also redefine the role of pathologists to experts able to agglomerate and interpret genetic/molecular, morphological, and imaging information to produce a more integrated and accurate diagnosis [92]. In this context, DNNs could also be trained to predict response to specific treatments and could be of help in devising patient stratification strategies for the optimization of clinical trials. Similarly, training DNNs with images from known responders and nonresponders to specific treatments (e.g., immunotherapy) may help better stratify patients for appropriate future personalized and precision-based clinical trials. The main issue with the use of DNNs is the need for a large number of manually curated cases in order to properly tune the hyperparameters of the network. Unification of criteria in multicentric data collection, as is under way (for example) in large worldwide efforts like the Human Connectome Project or the Alzheimer's Disease Neuroimaging Initiative, is therefore an important next step also in the field of pathology. Also, given the ever-rising importance of quality assurance in the nascent field of precision oncology, a centralized, DNN-based system could function as a quick and reliable tool for pathologists to request and receive second opinions as well as consensus in an extremely time-saving and cost-efficient manner.

## 6. Conclusion

The scientific dissertation reported here highlights the possible positive interaction among medical disciplines that base their activity on acquisition and interpretation of cancer imaging. Furthermore, the successful collaboration between diagnostic imaging and anatomic pathology departments can represent a fundamental step in the “tortuous” path to personalized medicine.

## Abbreviations

ADC: Apparent diffusion coefficient
AI: Artificial intelligence
BOLCs: Breast osteoblast-like cells
CT: Computed tomography
DNA: Deoxyribonucleic acid
DNN: Deep neural network
ER: Estrogen receptor
FDG: Fluorodeoxyglucose
MRI: Magnetic resonance imaging
OLCs: Osteoblast-like cells
PET: Positron-emission tomography
PR: Progesterone receptor
PSA: Prostate-specific antigen
PSMA: Prostate-specific membrane antigen
SPECT: Single-photon-emission computed tomography
TN: Triple-negative
TNM: Tumour-node-metastasis
US: Ultrasound
WSI: Whole slide imaging.
